# Molecular epidemiology of aquatic environments: challenges from sampling to implementation of surveillance programs

**DOI:** 10.3389/fpubh.2025.1652535

**Published:** 2025-10-01

**Authors:** Bradd Mendoza-Guido, Jose R. Montiel-Mora, Cristina Ureña-Salazar, Kenia Barrantes, Luz Chacón

**Affiliations:** Instituto de Investigaciones en Salud (INISA), Universidad de Costa Rica, San José, Costa Rica

**Keywords:** ARGS, public health surveillance, resource-limited settings, PCR-based detection, waterborne pathogens, environmental monitoring

## Abstract

Pathogens are introduced into wastewater through human and animal fecal discharge, ultimately contaminating aquatic environments such as rivers and beaches. Molecular tools are commonly used to track outbreak-related pathogens in wastewater due to numerous advantages such as enhanced sensitivity, speed, and specificity. However, many low- and middle-income countries (LMICs) face challenges in developing adequate sanitation infrastructure and accessing or implementing high-cost technologies, which hampers the integration of environmental surveillance into national and regional public health programs. This mini-review summarizes key challenges in applying molecular techniques for water-based epidemiological monitoring of waterborne pathogens in resource-limited settings. We examine obstacles related to sampling aquatic environments, including collecting samples from rivers and concentrating analytes from complex matrices such as wastewater and polluted river or beach waters, emphasizing the importance of preserving environmental representativeness. We provide a brief overview of the most widely used PCR-based technologies for detecting waterborne pathogens and antimicrobial resistance genes (ARGs), discussing their advantages and limitations. We also examine advanced high-throughput technologies, often inaccessible in LMICs, and emerging portable tools that may enhance detection where laboratory infrastructure is limited. Finally, through applied examples, we show how environmental data can make pathogen surveillance more accessible while bridging laboratory research with public health practice.

## Graphical abstract

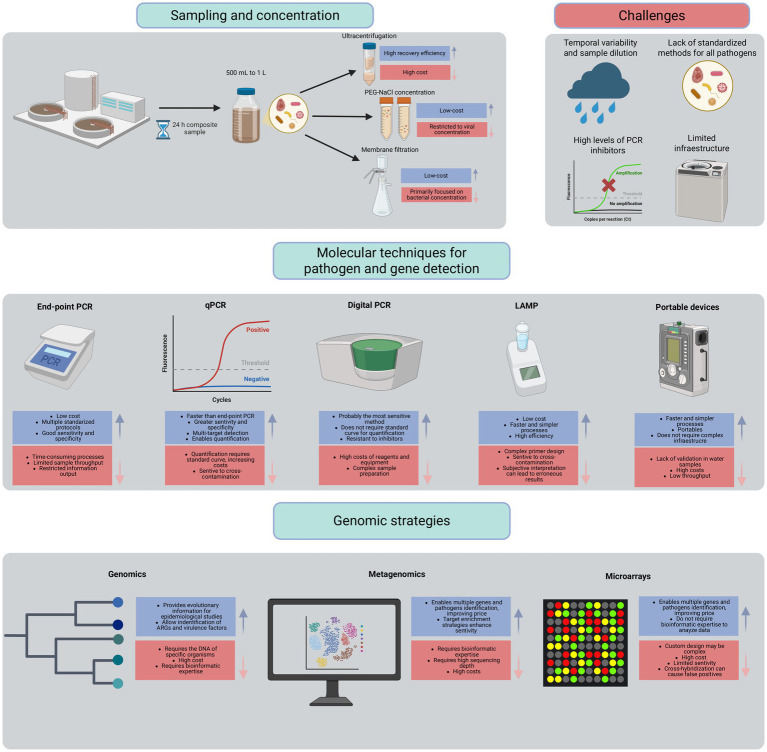

## 1 Introduction

Water consumption is essential for maintaining good health; however, in 2020 nearly 2 billion people lacked access to safely managed drinking water services, including hundreds of millions relying on unimproved sources or even surface water (1). This challenge is further compounded by the growing release of anthropogenic compounds, such as surfactants, hormones, antibiotics, and other pollutants, into aquatic environments (2, 3). Thus, contaminated ecosystems can serve as reservoirs and dissemination hubs for human pathogens and urban pollutants that spread through water bodies (4).

Several pathogens including bacteria, fungi, viruses and protozoan can be found in aquatic environments, leading to waterborne outbreaks of numerous diseases (5). These microorganisms are often released into wastewater through direct human and animal fecal discharge, which eventually flows into aquatic environments like rivers and beaches. Wastewater treatment plants (WWTPs) were designed to reduce suspended solids, organic matter and other contaminants harmful to public and ecosystem health. However, most WWTPs fail to significantly reduce microbiological loads, as disinfection steps are often omitted unless the wastewater is designated for regeneration (6).

Consequently, WWTPs have emerged as reservoirs that mediate the propagation of microorganisms, particularly antibiotic-resistant bacteria. The high-pressure environment created by the presence of contaminants such as antimicrobials, fosters the selection and retention of adaptive traits, such as antibiotic resistance genes (ARGs) (7, 8). Moreover, wastewater-based surveillance has been utilized to track many outbreak-related pathogens (including pathogenic viral strains) under different technologies, particularly molecular biology tools (9). Nevertheless, the integration of such strategies into governmental surveillance programs remains limited, particularly in LMICs where resources are scarce.

This review addresses key challenges in applying molecular techniques for waterborne pathogen surveillance in resource-limited regions. We also emphasize the importance of integrating molecular epidemiology strategies, framed within the One Health approach, into community and regional monitoring programs to mitigate pathogen spread and protect human health.

## 2 Sampling methods in aquatic environments

Molecular surveillance of waterborne pathogens requires robust sampling strategies tailored to the aquatic environment. Therefore, environmental representativeness and the integrity of genetic material are essential for obtaining reliable results. Factors such as matrix type, spatial and temporal variability, and the presence of inhibitors can influence sample quality and the accuracy of molecular analyses (10–13). Consequently, key steps include appropriate sample collection and preservation, effective concentration of target microorganisms, and addressing challenges that may hinder the detection of pathogens in water (14, 15).

### 2.1 Preparation of samples

Obtaining a representative water sample is a critical first step in molecular epidemiology. Sampling strategies must account for the temporal and spatial variability of aquatic systems (16–18). Grab samples, which are discrete samples collected at a single point in time, are simple and widely used. For instance, the World Health Organization recommends collecting at least 500 mL of grab samples from wastewater for poliovirus surveillance (19). However, short-duration grab samples may miss intermittent pathogen shedding events (20–22). To improve environmental representativeness, composite sampling is often employed by combining subsamples over 24 h or across multiple sites to capture fluctuations in flow and contamination levels (23, 24). In wastewater surveillance, 24-h composite samples collected using automated samplers provide more stable estimates of pathogen load than random grab samples (25). Nonetheless, all samples must be collected in sterile containers and kept at low temperatures until processed to prevent degradation of genetic material (26–28). Processing within 24–48 h or the addition of stabilizing agents, such as acidic pH buffers or RNA preservatives, is also recommended to prevent the degradation of pathogen nucleic acids, particularly in settings where immediate processing is not feasible (13, 29).

### 2.2 Filtration and concentration

Detecting waterborne pathogens often requires the processing of large water volumes, as these microorganisms are typically present in low concentrations (30). Therefore, several techniques have been developed to concentrate and filter microorganisms into smaller volumes (19). For instance, the most used methods for viruses include electropositive/electronegative filtration and ultrafiltration (31, 32). Among ultrafiltration approaches, hollow-fiber devices are capable of simultaneously recovering viruses, bacteria, and protozoa, with reported recovery efficiencies ranging from 70 to 90% for bacteria and protozoan oocysts (30, 33). However, one of their main disadvantages compared to other methods is their high cost (34).

Alternatively, precipitation-based methods have also been widely employed, such as the two-phase polyethylene glycol (PEG) flocculation protocol, recommended by the World Health Organization for the concentration of enteric viruses in wastewater (19, 35). In this approach, viruses are precipitated using PEG and salt, then resuspended in a small volume for downstream analysis (36). Similarly, aluminum hydroxide adsorption–precipitation and glycine beef extract elution are also used in specific protocols to concentrate viruses from environmental waters (37, 38). For bacterial pathogens, membrane filtration (e.g., 0.45 μm pore size filters) is routinely applied: microbes are retained on the membrane, which can then be used directly for DNA/RNA extraction or culture-based methods (39, 40).

### 2.3 Challenges in monitoring aquatic environments

One of the primary challenges in environmental surveillance is the spatial and temporal variability of pathogen presence, which can lead to false negatives if the sampling is not representative (41, 42). Furthermore, rainfall variability poses another challenge, as it can have direct and indirect effects on DNA detection, primarily by influencing sample dilution. Heavy rains can increase the water volume, followed by dilution of the DNA present in the environment sampled (43, 44). Additionally, there is no universal method capable of recovering all types of pathogens from water samples (12), primarily because viruses, bacteria, and protozoa differ in size and physicochemical properties, so protocols effective for one group may be inefficient for others. Moreover, filtration or elution processes often result in partial loss of pathogens, further compromising detection (45).

Environmental water samples also contain natural inhibitors, such as humic substances, fulvic acids, and phenolic compounds, that can interfere with PCR reactions, reducing the sensitivity of molecular detection methods (12). A significant limitation of molecular techniques like qPCR is their inability to differentiate between viable pathogens and residual nucleic acids from non-viable organisms, which complicates accurate risk assessment (46).

In LMICs, limited infrastructure, insufficient technical training, and restricted laboratory access further hinder the systematic implementation of these methods. Consequently, low-cost approaches with streamlined protocols should be prioritized to support continuous surveillance and produce actionable data for public health decision-making (47, 48).

## 3 PCR and LAMP methods for pathogens and target genes detection

Since the 1990s, polymerase chain reaction (PCR) has been widely adopted to amplify and detect viral and bacterial DNA in water samples, offering key advantages such as enhanced detection limits, reduced processing time and cost, and the ability to identify a broad range of microorganisms (10). Its high sensitivity, along with its specificity and reliability (12, 13), has established PCR as a cornerstone technique in molecular epidemiology studies of waterborne pathogens. Moreover, thermal cyclers, essential instruments for PCR, are now standard equipment in most molecular biology laboratories, including those in many LMICs.

End-point PCR has long been regarded as the gold standard for detecting various pathogens, including viruses and bacteria. Viral detection by conventional methods is often complex, typically requiring the concentration of viral particles and propagation in permissive host cells (10). In contrast, bacterial pathogens are generally easier to culture; however, traditional culture-based methods can be limited by difficulties in species-level identification and the presence of viable but non-culturable strains in environmental samples, increasing both the complexity and cost of detection (10). These limitations have made PCR-based methods highly valuable for accurate pathogen identification.

Although end-point PCR remains widely used, quantitative PCR (qPCR) has gained increasing traction due to its superior sensitivity, faster turnaround time, and its ability to simultaneously amplify, detect, and quantify specific nucleic acids. This enables more reproducible and timely assessments that support prompt public health interventions (14). qPCR works by detecting fluorescence emitted during DNA amplification, which correlates with the quantity of target DNA present in the sample. Fluorescence can be generated through intercalating dyes, such as SYBR Green, or through labeled probes containing a fluorescent reporter molecule that binds specifically to the target DNA (15).

Using qPCR can be helpful in wastewater-based surveillance, which plays an important role in early detection of diseases. Since the COVID-19 pandemic, this area of research has gained popularity due to its potential to detect prospective cases or outbreaks. A 2020 study conducted by a research group in Córdoba, Argentina, employed qPCR to detect SARS-CoV-2 genetic material in wastewater samples from multiple sites throughout the city. The findings of the study suggest that this monitoring approach is effective as an early warning system for future outbreaks in regions with a stable resident population. Conversely, in areas with low population density or significant population flux, such as those influenced by tourism, its utility may be limited to confirming the presence of the disease in the area (49). It is important to note that for wastewater-based surveillance to be effective, a thorough understanding of the sewer network’s infrastructure and the specific populations contributing to the sampled wastewater is essential. This requirement may pose a significant challenge in LMICs, where such information may be incomplete, inaccurate, or difficult to access (50).

Another PCR-based technique with high potential for use in molecular epidemiology is digital PCR (dPCR) (51, 52). Like qPCR, it relies on the detection of fluorescence resulting from DNA amplification. However, dPCR differs by partitioning the sample into thousands of individual reactions, each ideally containing a single DNA molecule. This allows for the absolute quantification of the initial DNA fragments with greater accuracy and sensitivity. In addition, dPCR has been reported to offer greater tolerance to inhibitors present in complex samples, which is particularly relevant for the implementation of DNA quantification methods in water samples (52). However, dPCR requires expensive reagents that are often not readily available in LMICs. These costs are further increased by importation taxes and shipping fees. Therefore, implementing dPCR in epidemiological programs using environmental samples in LMICs will require optimization of both reagent and equipment costs.

Finally, loop-mediated isothermal amplification (LAMP) is a variation of the PCR that does not require a thermal cycler instrument, as the entire process occurs at a constant temperature. It represents a promising alternative that is faster, simpler, and more accessible than conventional PCR methods. LAMP has been proposed as a low-cost option for the rapid identification of multiple pathogens (53); although several limitations remain.

LAMP requires the design of 4 to 6 primers targeting specific regions within a short DNA segment, which makes primer design complex. Additionally, due to the high efficiency of LAMP, the risk of false positives from contamination is considerable with improper handling. Furthermore, the turbidity- and colorimetric-based detection of LAMP-positive reactions is subjective, which may increase the likelihood of erroneous results (54, 55). Thus, although the method is promising, it will require further optimization and validation for its application in molecular epidemiology of environmental samples.

PCR-based methods have a limited ability to distinguish between pathogen variants/genotypes and to provide evolutionary insights, both of which are essential for tracking epidemiological patterns and understanding genetic flow. Although some qPCR probes have been developed to detect specific variants of pathogens such as SARS-CoV-2 (56, 57) and norovirus (58), the short length of these probes constrains their resolution. As a result, findings from such assays often require further confirmation using genetic/genomic data.

## 4 Genomic and metagenomic methods

Next,-generation sequencing (NGS) methods have been proposed as a promising solution for pathogen and antimicrobial resistance (AMR) surveillance (59, 60), particularly because genomic data can reveal critical evolutionary and epidemiological insights into outbreak dynamics, information beyond the reach of conventional PCR-based methods. Although the COVID-19 pandemic highlighted the urgency of implementing such methodologies, the capacity to apply NGS for genomic surveillance of environmental samples remains limited in LMICs due to budgetary constraints (61). Additionally, most programs prioritize the implementation of genomic surveillance in clinical settings, but fewer efforts allocate resources to the investigation of environmental samples (62).

Furthermore, the implementation of metagenomic approaches for genomic surveillance has been strongly encouraged (63, 64). Such techniques can improve the cost-efficiency per sample, as sufficient DNA quantity and sequencing depth allow the simultaneous detection of a wide range of pathogens and their effectors (virulence and ARGs). Similarly, microarrays offer a promising solution for multi-target detection by enabling the identification of hundreds of targets with high specificity through well-designed probes. For example, a DNA microarray was recently developed in Mexico to identify 252 etiological agents (with 38,000 probes) from environmental samples (65).

Following the SARS-CoV-2 pandemic, genomic surveillance has been increasingly implemented in LMIC to investigate epidemiological patterns of pathogens in water samples. For example, the co-circulation and abundance SARS-CoV-2 variants in wastewaters were monitored in Uruguay; serving as a complementary tool for tracking community-level transmission and informing early public health decisions (66). Building on this approach, genomic technologies have been adapted to study other pathogens beyond SARS-CoV-2. Also in Uruguay, a recent study applied wastewater-based genomic surveillance using targeted enrichment sequencing to monitor 42 respiratory viruses. They detected several pathogens that had not been previously reported in circulation (67).

While these technologies offer great potential, their high costs and the need for specialized bioinformatics expertise remain significant barriers, potentially limiting their accessibility in LMICs. These resource limitations underscore the need to establish region-wide networks that efficiently standardize multi-pathogen sequencing centers, facilitating the integration of genomic surveillance into regional public health policies (60).

## 5 Portable diagnostics and their impact on rapid response in LMICs

Portable diagnostic devices have been proposed as innovative tools for the rapid detection of infectious pathogens. These include point-of-care and molecular tests that enable the timely identification of pathogens and prompt treatment. Most of these portable devices have been validated using clinical samples (such as blood, urine, saliva, and other fluids), but some have even been tested on environmental water samples, as reviewed by Kumar et al. (68) and Oon et al. (62). These systems primarily rely on the detection of nucleic acids, proteins, or specific cell features of pathogens using biosensors and microfluidic systems.

The concept behind these devices is promising, especially for pathogen detection in areas where high-tech laboratories and trained personnel are unavailable, and where sample transportation can take several days. Although many of these devices have improved their sensitivity and specificity (62), they still face challenges when dealing with complex matrices such as environmental water samples, which often contain inhibitors. Moreover, compared to the previously mentioned gold standard methods, these devices have yet to be widely implemented or validated in surveillance programs. In addition, the cost per unit remains high, presenting a financial barrier in LMICs settings. While these tools offer promising solutions for rapid diagnostics, they simultaneously underscore the persistent economic constraints faced by resource-limited settings. Accordingly, the development and prioritization of low-cost, portable devices with validated protocols are essential to support sustained surveillance efforts and generate actionable data for informed public health decision-making (69).

## 6 Environmental and public health implications of molecular epidemiology of aquatic environments

Since its initial implementation for polio surveillance, environmental monitoring has been applied across multiple contexts to support public health efforts. Wastewater-based surveillance programs have demonstrated their effectiveness as early warning systems, helping to mitigate pathogen transmission and enabling the estimation of infection trends within populations (70).

Moreover, environmental surveillance of AMR offers a valuable opportunity to strengthen the One Health approach (71). This type of surveillance enables the identification of multidrug-resistant microorganisms, through the detection of resistance genes/markers, and also the potential environmental reservoirs of antimicrobial resistance genes (ARGs). Such information is critical to guide evidence-based decision-making regarding the prudent use of antimicrobials in human and veterinary medicine, as well as in industrial applications (72, 73).

Multiple programs for the surveillance of microorganisms and antimicrobial resistance genes (ARGs) are now operating worldwide. In the United States, the Centers for Disease Control and Prevention (CDC) has led the National Wastewater Surveillance System (NWSS) (74) since 2020, monitoring SARS-CoV-2, influenza viruses, respiratory syncytial virus (RSV), mpox virus (MPXV), and ARGs across more than 1,500 sentinel sites, covering approximately 45% of the U.S. population.

In Europe, the Sewage Sentinel System (EU4S) (75), coordinated by the European Commission, monitors SARS-CoV-2, RSV, influenza viruses, and antimicrobial resistance (AMR) across 11 EU member states, with more than 1 million measurements collected. Furthermore, in Asia, the Mekong Basin Disease Surveillance (MBDS) (76), network, involving Cambodia, China, Laos, Myanmar, Thailand, and Vietnam, implements cross-border surveillance efforts, including wastewater monitoring for priority infectious diseases such as dengue, malaria, influenza, cholera, and tuberculosis. Likewise, in Oceania, Australia launched the National Wastewater Surveillance Program (77) in 2025 to monitor SARS-CoV-2, influenza viruses, RSV, poliovirus, mpox virus, and Japanese encephalitis virus across the entire country.

However, while comprehensive surveillance systems have been established in many high-income countries, efforts in Latin America, Africa, and other low- and middle-income regions remain largely limited to pilot projects or short-term studies, often constrained by funding, infrastructure, and technical capacity (71). Given the proven benefits of environmental surveillance for both pandemic containment and AMR mitigation, there is an urgent need to promote its global expansion and ensure that all countries, regardless of income level, have access to the tools and capacities required to implement sustainable, integrated surveillance programs.

## 7 Conclusion

- Molecular epidemiology has significantly advanced the detection and monitoring of waterborne pathogens and AMR in aquatic environments. However, several challenges remain, particularly in resource-limited settings.
- Robust and representative sampling strategies are essential to avoid false negatives and ensure nucleic acid integrity.
- Among molecular techniques, PCR and qPCR remain the most widely used due to their sensitivity, reproducibility, and relative accessibility. dPCR offers improved quantification accuracy but still requires cost reduction, while the LAMP technique shows promising field applicability and low cost, though it needs further optimization.
- Genomic and metagenomic approaches provide valuable information on pathogen diversity, evolutionary traits, and ARGs, which are highly important for epidemiological programs. However, their high costs and limited infrastructure in LMICs restrict their widespread use, especially in non-clinical samples.
- Wastewater-based surveillance and epidemiology are valuable early warning tools for outbreaks, but their integration into public health systems remains limited, especially in LMICs. Expanding their impact requires affordable, validated diagnostics, regional collaboration, and alignment with One Health strategies, ensuring that environmental data can be translated into timely public health interventions and to improve preparedness, especially in vulnerable regions.
